# Osteogenesis of peripheral blood mesenchymal stem cells in self assembling peptide nanofiber for healing critical size calvarial bony defect

**DOI:** 10.1038/srep16681

**Published:** 2015-11-16

**Authors:** Guofeng Wu, Mengjie Pan, Xianghai Wang, Jinkun Wen, Shangtao Cao, Zhenlin Li, Yuanyuan Li, Changhui Qian, Zhongying Liu, Wutian Wu, Lixin Zhu, Jiasong Guo

**Affiliations:** 1 Department of Histology and Embryology, Southern Medical University, Guangzhou 510515, China; 2 Department of Orthopaedics, Zhujiang Hospital, Southern Medical University, Guangzhou 510280, China; 3 Key Laboratory of Tissue Construction and Detection of Guangdong Province, Guangzhou 510515, China; 4 Institute of Bone Biology, Academy of Orthopaedics, Guangdong Province, Guangzhou 510665, China; 5 Department of Anatomy, Li Ka Shing Faculty of Medicine, The University of Hong Kong, Pokfulam, Hong Kong SAR, China; 6 State Key Laboratory of Brain and Cognitive Sciences, Li Ka Shing Faculty of Medicine, The University of Hong Kong, Pokfulam, Hong Kong SAR, China; 7 GHM Institute of CNS Regeneration, Jinan University, Guangzhou 510632, China; 8 Guangdong Institutes of Biomedicine and health, Chinese Academy of Sciences, Guangzhou 510530, China

## Abstract

Peripheral blood mesenchymal stem cells (PBMSCs) may be easily harvested from patients, permitting autologous grafts for bone tissue engineering in the future. However, the PBMSC’s capabilities of survival, osteogenesis and production of new bone matrix in the defect area are still unclear. Herein, PBMSCs were seeded into a nanofiber scaffold of self-assembling peptide (SAP) and cultured in osteogenic medium. The results indicated SAP can serve as a promising scaffold for PBMSCs survival and osteogenic differentiation in 3D conditions. Furthermore, the SAP seeded with the induced PBMSCs was splinted by two membranes of poly(lactic)-glycolic acid (PLGA) to fabricate a composited scaffold which was then used to repair a critical-size calvarial bone defect model in rat. Twelve weeks later the defect healing and mineralization were assessed by H&E staining and microcomputerized tomography (micro-CT). The osteogenesis and new bone formation of grafted cells in the scaffold were evaluated by immunohistochemistry. To our knowledge this is the first report with solid evidence demonstrating PBMSCs can survive in the bone defect area and directly contribute to new bone formation. Moreover, the present data also indicated the tissue engineering with PBMSCs/SAP/PLGA scaffold can serve as a novel prospective strategy for healing large size cranial defects.

Calvarial bony defects may result from congenital defects, traumatic injuries, neurosurgical procedures or tumor resections^1^. For both aesthetic and functional reasons, successful healing of the defects is quite important for the subjected patients, which still remains a major concern for clinical and basic researchers. Due to the drawbacks of limited sources, immunologic rejection, and disease transmission, the natural grafts such as autologous, allogeneic and xenogeneic bone grafts are impossible to be used as routine items in clinic, even though have good osteogenic potential^2^,^3^.

During the past years tissue engineering has been regarded as a substitute for natural source bone grafts^4^,^5^. The first important aspect of tissue engineering is obtaining suitable seed cells. Mesenchymal stem cells (MSCs) may serve as a plausible candidate since its capabilities of multipotential differentiation include osteogenesis^6^. MSCs were first identified and isolated from bone marrow^7^ and then found in various tissues including umbilical cord^8^, adipose tissue^9^, and peripheral blood^10^. Among these sources, the peripheral blood MSCs (PBMSCs) draw increasing attention as they share similar biological characteristics with MSCs derived from bone marrow or adipose tissue^11^,^12^. PBMSCs permit an autologous low-cost source of stem cells which may be easily harvested from patient’s blood by non-invasive procedures. Although two previous reports already utilized PBMSCs for bony tissue engineering with positive outcomes^13^,^14^, there is still no solid evidence to demonstrate the PBMSC’s capabilities of survival and osteogenesis in the bone defected area. Therfore, the first question we aimed to address is whether PBMSC has potential to be used as seed cells for bony tissue engineering.

The other important aspect of stem cell-based bone tissue engineering is developing an efficient scaffold to reconstruct the defect as well as support the survival and osteogenesis of the grafted stem cells. In previous reports related to this field, the stem cells were always seeded in scaffold of inorganic macromolecular such as calcium phosphate^15^,^16^, hydroxyapatite^17^,^18^, or synthetic polymers such as poly(lactic)-glycolic acid (PLGA)^9^,^19^,^20^. Of course these materials have good physical properties to reconstruct the bone defect, however, they cannot provide true three dimensional microenvironment for the seeding cells, as the porosity in these scaffolds is much larger than the size of cells^21^. Herein, we aimed to design a novel scaffold to deliver PBMSCs and repair calvarial bone defect. For this reason PBMSCs were seeded into a three dimensional (3D) nanofiber scaffold of self-assembling peptide (SAP) and cultured in osteogenic inductive medium. As it is well known, SAP can mimic natural extracellular matrix to deliver different kinds of cells in tissue engineering^22^,^23^,^24^. Our data obtained from the current study also illustrated PBMSCs could survive and produce osteogenic differentiation in the scaffold, both *in vitro* and *in vivo*. Due to SAP scaffold presenting as hydrogel, which is not suitable for reconstructing large scale calvarial bone defect, the SAP seeded with PBMSCs was splinted by two PLGA membranes to fabricate a sandwich-like scaffold. The PBMSCs/SAP/PLGA composite scaffold was transplanted to repair a critical-size calvarial bone defect in rats. By this model we first confirmed that PBMSCs could survive and provide osteogenesis after being implanted into the bone defect area and could directly contribute to new bone formation. Also, the present study indicated tissue engineering with PBMSCs/SAP/PLGA is a novel prospective strategy for healing large size calvarial bone defects.

## Methods

### Ethics Statement

All animal experiments, including surgery and tissue collection, were carried out with the approval of the Southern Medical University Animal Care and Use Committee in accordance with the guidelines for the ethical treatment of animals. All efforts were made to minimize animal suffering.

### Isolation of peripheral blood derived mesenchymal stem cells (PBMSCs)

The peripheral blood was harvested from green fluorescence protein (GFP) transgenic Sprague-Dawley rats (“green rat CZ-004” SD TgN(act-EGFP) OsbCZ-004), aged 4 weeks, and diluted immediately with heparinized saline in a 1:1 proportion. The diluted blood was gently loaded onto Ficoll density gradient (Sigma) in 10 mL tubes and centrifuged for 25 min at 1600 g. Mononuclear cell fraction was collected and rinsed twice with Hank’s Balanced Salt Solution (HBSS), and then cultured at 10^6^ cells/cm^2^ in DMEM/F12 medium supplemented with 10% fetal bovine serum (FBS). The medium was replaced every three days and non-adherent cells were discarded. After the primary culture reached approximately 80% confluence, cells were passaged regularly and the 5^th^ passaged (P5) cells were used for further experiments in this project.

### Flow cytometric analysis of the immunophenotyping of PBMSCs

The following antibodies were used to perform flow cytometric analysis on P5 PBMSCs: phycoerythrin (PE) conjugated hamster anti-CD29, mouse anti-CD45, mouse anti- CD90 and isotype control hamster or mouse IgG (BioLegend); non-conjugated mouse anti-CD105, rabbit anti-CD44 and rabbit anti-CD146 (Abcam); Alexa 568-goat anti-mouse or rabbit secondary antibody IgG (Molecular Probe). For CD29, CD45 and CD90 assaying, the harvested P5 PBMSCs were washed with cold phosphate buffered saline (PBS), blocked with 1% bovine serum albumin (BSA), and then incubated with phycoerythrin (PE) conjugated antibodies at 4 °C for 30 min. Isotype IgGs were used to replace specific antibodies as control for nonspecific staining. For CD44, CD105 and CD146 assessing, the cells were incubated with non-conjugated first antibodies at 4 °C overnight after be blocked with 1%BSA. Then, the cells were treated with Alexa 568-goat anti-mouse or rabbit secondary antibody IgG for 2 hour at room temperature. Meanwhile, the first antibodies were replaced by PBS for negative control. All cells were analyzed on a FACScan flow cytometer (BD FACSCalibur) by using the CellQuest software.

### Multilineage differentiation potential evaluated by *in vitro* chemical induction

P5 PBMSCs were seeded onto the coverslips in 1 × 10^4^ cells/coverslip, and cultured within 24-well plates. The cells were cultured in basic medium for 12 hours and then subjected to a series of chemically inductive differentiation protocols as follows.

For osteogenesis, the cultures were induced with αMEM medium containing 10% FBS, 10^−8^ M dexamethasone, 10 mM β-glycerophosphate, 50 μM L-ascorbic acid-2-phosphate, and 5 × 10^−8^ M 1α,25-Dihydroxyvitamin D3. Twenty-one days later alkaline phosphatase (ALP) staining, von kossa staining, alizarin red staining, as well as osteocalcin immunostaining were performed to evaluate osteogenic products as previously described^25^,^26^,^27^.

For chondrogenesis, cells were induced for 21 days in DMEM with 10% FBS, 10^−7^ M dexamethasone, 50 μM L-ascorbic acid-2-phosphate, and 10 ng/ml transforming growth factor-β, 1% insulin-transferrin-selenium, 5 mM sodium pyruvate, 40 μg/ml L-proline, 1% non-essential amino acid. The differentiation was evaluated by routine Toluidine blue staining^28^.

For adipogenesis, cells were cultured for 21 days in DMEM with 10% FBS, 0.5 mM isobutylmethylxanthine, 0.5 μM hydrocortisone, 10 μg/ml insulin, 1 μM dexamethasone, 2 mM L-glutamine and 100 μM indomethacin. The formation of lipid vacuoles was assessed by Oil Red O staining^29^.

For neurogenesis, cells were induced in Neurobasal medium with 10 μM all trans-Retinoic Acid (ATRA), 20 ng/ml basic Fibroblast Growth Factor (bFGF), 50 μM forskolin, 5 IU/ml heparin, 20 ng/ml Brain-Derived Neurotrophic Factor (BDNF), 1% N2, 2% B27, and 5% FBS. Fourteen days later, immunocytochemistry with antibody of β-tubulin III was performed to monitor the differentiated neurons^30^.

For Schwann cell differentiation, cells were first treated with 1 mM β-Mercaptoethanol (β-ME) in Neurobasal medium for 24 h then incubated with 350 ng/ml ATRA for 3 days, followed by 21 day induction with 10 ng/ml bFGF, 12.5 μM forskolin, 10 ng/ml heregulin, and 5 ng/ml Platelet-Derived Growth Factor (PDNF). Finally, the cell differentiation was identified using immunocytochemistry with antibody of Schwann cells specific maker S-100^23^.

### *In vitro* osteogenesis of PBMSCs in a three dimensional (3D) scaffold of self-assembling peptide

In the current study RADA16-I peptide (BD Biosciences), which is one of type I self-assembling peptides (SAPs), was used to carry PBMSCs for further transplantation. This peptide can be dissolved in deionized water; once contacted with culture medium, it spontaneously self-assembles into a 3D nanofibers scaffold with greater than 99% water content, which is similar to native extracellular matrix^21^,^31^.

In order to figure out whether PBMSCs can survive and perform osteogenesis within this scaffold, an *in vitro* test was done before transplantation. 1 × 10^4^ P5 PBMSCs in 5 μL DMEM medium were rapidly mixed with 50 μL 1% SAP solution. The mixture was gently and quickly plated to a dish with osteogenic medium (αMEM supplemented with10% FBS, 10^−8^ M dexamethasone,10 mM β-glycerophosphate, 50 μM L-ascorbic acid-2-phosphate, and 5 × 10^−8^ M 1α,25-Dihydroxyvitamin D3). Twenty-one days later the culture was fixed with 4% paraformaldehyde for 2 hours, cryoprotected in 30% sucrose in PBS for 12 hours, embedded in optimum cutting temperature compound (OCT), then sectioned(50 μm) and mounted onto poly-l-lysine subbed slides. Some slides were used to do ALP staining while the remaining were immumostained with the antibodies of osteocalcin and chondroitin sulphate proteoglycans (CSPG).

### Scaffold fabrication for calvarial bone tissue engineering

For further tissue engineering repair the calvarial bone defect, a novel composite scaffold was fabricated with PBMSCs, SAP and membranes PLGA. The main procedures are as follows.

The preparation of PLGA membrane with a thickness of 50 μm has been described previously^32^,^33^ with minor modifications. Briefly, 0.5 g PLGA (PLA:PGA = 85:15) was dissolved in 10 g dichloromethane with 0.5 g NaCl particles (diameter of 75–95 μm) added into the solution. The solution was mixed quickly and poured into a Teflon-coated horizontal mold. After evaporation of the solvent in a fume hood for 48 hours, the formed membrane was washed with water thoroughly to remove the NaCl particles. The membrane was then cut into disc-shapes with a diameter of 8 mm, immersed in 75% alcohol overnight for sterilization, and rinsed three times with saline.

PBMSCs/SAP scaffold was prepared as follows. 2 × 10^5^ P5 PBMSCs in 10 μL DMEM medium were rapidly mixed with 90 μL 1% SAP solution. The mixture was gently and quickly plated to a coverslip which was placed in a 35 mm culture dish. Then, 800 μL osteogenic medium was added to the dish. Just before the mixture assembled into gel-like scaffold, another coverslip was covered on the scaffold with controlled pressure to planarize the dome to the level of the surface of culture medium. In this way, the thickness of scaffold was controlled at ~1 mm. Then 1 ml osteogenic medium was added into the culture dish after the upper coverslip was carefully removed. Seven days after osteogenic induction, 10 μg/mL BrdU was added to the medium for another 48 hours culture to label the cells. BrdU is conducive to distinguish the grafted cells and host cells after transplantation.

**Figure 1:**
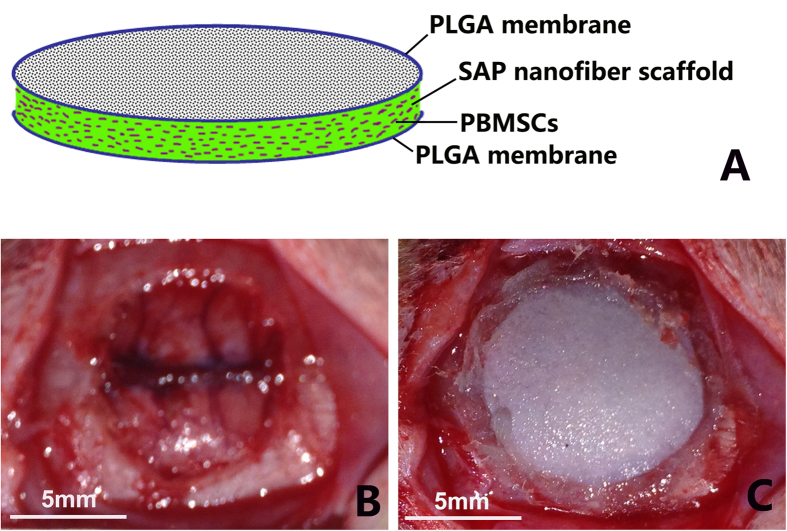
Tissue engineering and surgery. (**A**) a schematic illustration showing the fabricated sandwich-like PBMSCs/SAP/PLGA scaffold for transplantation. (**B**) a critical size defect (8 mm in diameter) was created in the middle of cranial bones in rat. (**C**) a PMBSCs/SAP/PLGA scaffold was implanted into the defect area.

Immediately before implantation, a sandwich-like PBMSCs/SAP/PLGA scaffold was fabricated as described by the schematic illustration in Fig. 1A. The prepared PBMSCs/SAP gel-like scaffold with bottom coverslip was taken out from the culture dish. A PLGA membrane was covered onto the scaffold, then all the materials were turned over together. After this a PLGA membrane was on the bottom side of the scaffold and the coverslip was on the upper side. The coverslip was then carefully removed and another PLGA membrane was covered onto the lamina of PBMSCs/SAP scaffold. Finally, the scaffold was trimmed to disc-shapes with a diameter of 8 mm.

### Surgical procedures

Adult male Sprague-Dawley rats (220–250 g, supplied by the Experimental Animal Center of Southern Medical University) were used for the surgery. The animals were anesthetized with 1% pentobarbital sodium (40 mg/kg, i.p.). Under sterile conditions a midline incision on the scalp was made to expose the parietal bones. A critical size defect (8 mm in diameter)^34^ was created in the middle of cranial bones by a sterile drill (Fig. 1B). The PMBSCs/SAP/PLGA scaffold, as described above, was implanted into the defected area. Tissue glue (3M Vetbond^TM^) was used to adhere the edge between graft and host (Fig. 1C). The incision was sutured layer by layer.

For a control a scaffold of SAP/PLGA was prepared the same way as PBMSCs/SAP/PLGA scaffold with the exception of replacing the PBMSCs suspension with same value of culture medium. This SAP/PLGA was then implanted by the same procedures as described previously.

### New bone formation detected by microcomputerized tomography and histology

Twelve weeks after surgery, calvarical samples (n = 8 for each group) were harvested and fixed in 4% paraformaldehyde for 48 hours. Mineral formation within the defect area was evaluated using microcomputerized tomography (Micro-CT, CT-80; ScanCo Medical, Bassersdorf, Switzerland) at a resolution of 25 μm, utilizing 55 kV and 145 μA with no aluminum filter. The bone volume (BV) in the defect area and the bone mineral density (BMD) of new formed bone were calculated using the Scanco analysis software.

After Micro-CT imaging the tissues were cut in half through the midline of implants and decalcified in 17% EDTA for 4 weeks at room temperature. The samples were dehydrated in grade ethanol, embedded in paraffin, cut into 5 μm thick sections, and then deparaffinized and processed for routine hematoxylin/eosin (H&E) staining. Image Pro Plus Software was used to quantify the newly formed bone as a ratio of the new formed bone area to the total defect area, as it has been described previously^8^. The outline of the implant area was traced and measured as the entire defect area. We then calculated the surface area of the new formed bone in the implant area.

### *In vivo* survival and osteogenesis of PBMSCs evaluated with immunohistochemistry

The rats (n = 5) implanted with PBMSCs/SAP/PLGA scaffold were transcardially perfused with 4% paraformaldehyde in 0.1 M phosphate buffer (pH 7.4) at 2 weeks post-surgery. The implants were carefully harvested from the bone defect area, then post fixed with 4% paraformaldehyde overnight, cryoprotected in 30% sucrose for 24 hours, and embedded in OCT. Cryostat sections (20 μm) were then cut and mounted onto poly-l-lysine subbed slides. Immunofluorescent staining with osteocalcin antibody was performed to detect the osteogenic differentiation of transplanted PBMSCs. The slices were incubated with primary antibody of mouse anti- osteocalcin overnight at 4 °C and treated with Alexa 568-goat anti-mouse secondary antibody IgG for 2 hours at room temperature. Images were captured by confocal microscope(Olympus F1-1000).

We also labeled the PBMSCs by BrdU before transplantation and traced them by BrdU immunohistochemistry at 12 weeks after transplantation. The prepared sections were denatured by boiling in citrate buffer (PH6.0) for 40 min, followed by incubating with 3% H_2_O_2_ for 15 min, 2N HCl for 30 min, Boric acid buffer (pH8.5) for 10 min, 0.1% trypsin and 1% bovine serum albumin for 30 min, respectively. The sections were then incubated with the primary antibody of monoclonal mouse anti-BrdU at 4 °C overnight and secondary antibody of goat anti-mouse IgG for 2 hours at room temperature. Finally, the sections were stained with 3,3′-Diaminobenzidine (DAB) for 5 min prior to nucleus counterstaining with hematoxylin.

### Statistical analyses

All data collected were expressed as mean ± standard deviation and statistical comparisons were assessed by Student’s paired t-test using SPSS 20.0 software. A P-value of less than 0.05 was considered statistically significant.

## Results

### Characterization of PBMSCs

**Figure 2:**
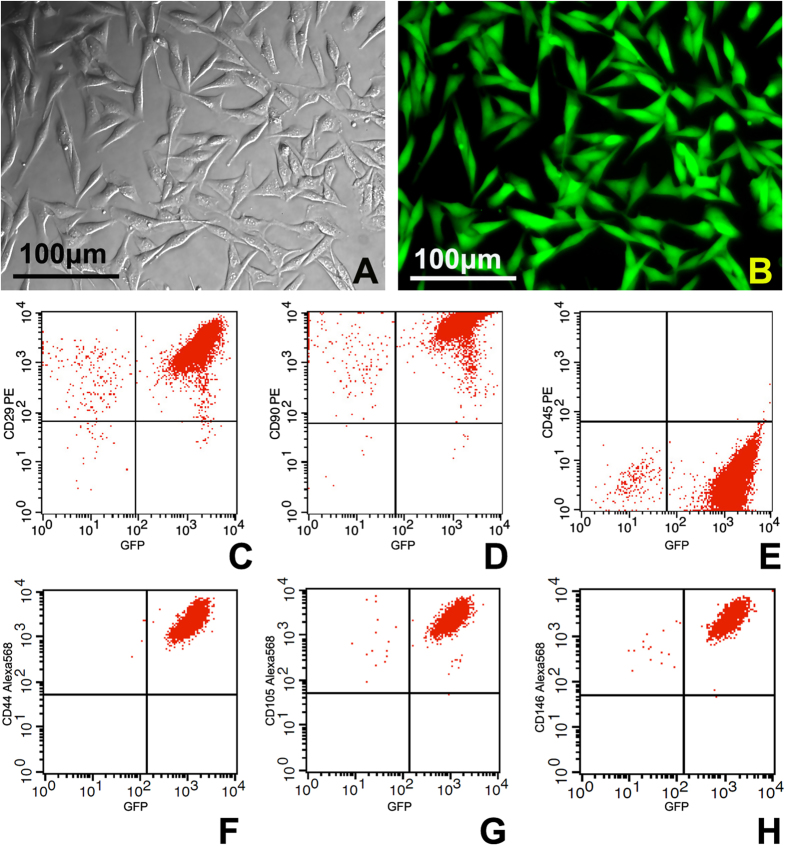
The morphology and immunophenotyping of PBMSCs. (**A,B**) phase contrast and fluorescent images of P5 PBMSCs isolated from GFP transgenic rats. (**C–H**) Immunophenotypic analyses by flow cytometry indicated P5 PBMSCs were strongly positive for mesenchymal lineage markers CD29d, CD44, CD90, CD105 and CD146 while negative for hematopoietic lineage marker CD45.

The freshly cultured PBMSCs grew slowly and appeared spindle shape after the initial 3~4 days. After the initial 3~4 days the PBMSCs grew more quickly and changed to typical polymorphic fibroblast-like morphology. By around 14 days the PBMSCs reached approximately 80% confluence. After being subcultured every 3 days, the cells appeared to be a relatively homogeneous morphology (Fig. 2A). As the cells were isolated from GFP transgenic rats, all cultures showed green fluorescence under fluorescent microscope (Fig. 2B).

Immunophenotypic analyses by flow cytometry indicated that the cells at P5 were strongly positive for mesenchymal lineage markers, CD29d (97.89% ± 5.24%) and CD90 (98.05% ± 6.19%), while negative for hematopoietic lineage marker CD 45 (0.07% ± 0.02%) (Fig. 2C–H).

### Multiple differentiation potential of PBMSCs

**Figure 3:**
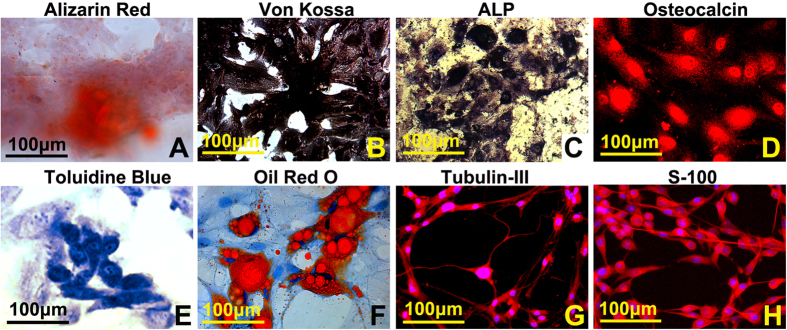
*In vitro* multilineage differentiation of PBMSCs. (**A,B**) Calcium deposits, orange-red color stained by alizarin red or black color stained by von kossa staining, were scattered in the osteogenic induced culture. (**C,D**) ALP and osteocalcin expression in the osteogenic cells were illustrated by ALP staining and immunocytochemistry. (**E**) aggrecan expressed in chondrogenesis induced cells was demonstrated by Toluidine blue staining. (**F**) lipid vacuoles in the adipogenesis induced cells were showed by Oil Red O staining. (**G,H**) induced neurons and Schwann cells were identified by immunocytochemistry with antibodies of β-tubulin III and S100.

After being incubated with designed specific chemical inductive media, the PBMSCs differentiated into osteoblast, chondroblast, adipocyte, neuron, and Schwann cell-like cells, respectively. In order to identify the differentiated cells, different staining protocols were performed as described in the Method section. The identifications of each goal-oriented cells are as follows. Calcium deposits, orange-red color stained by alizarin, red or black color stained by von kossa staining, were scattered in the osteogenic induced culture (Fig. 3A,B). ALP, which is an early marker for osseous differentiation and is widely used as osteoblast specific marker, was expressed in the induced cells in varying degrees (Fig. 3C). Additionally, immunocytochemistry and confocal imaging showed osteocalcin positive particles distributing in the osteogenic cells (Fig. 3D). In short, the osteo-differentiation was identified by four methods. Aggrecan expressed in chondrogenesis induced cells was demonstrated by Toluidine blue staining (Fig. 3E) while lipid vacuoles in the adipogenesis induced cells were showed by Oil Red O staining (Fig. 3F). When induced to differentiate into neurons or Schwann cells, these cells were immuno-positive for neuron’s specific maker of β-tubulin III or Schwann cell’s specific maker of S-100 (Fig. 3G,H).

### Self-assembling peptide provided a 3D scaffold for the survival and osteogenesis of PBMSCs

**Figure 4:**
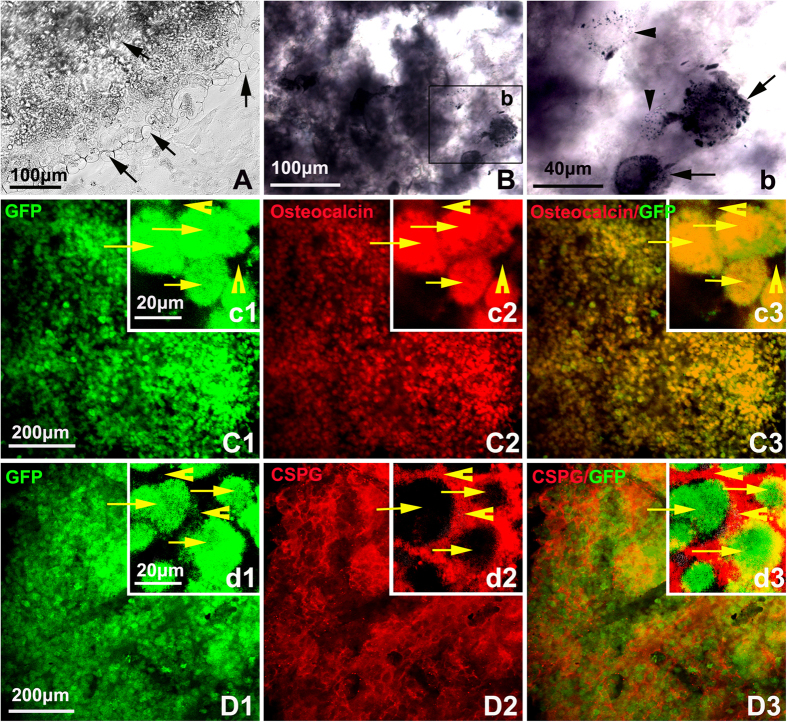
Survival and osteogenesis of PBMSCs in 3D scaffold of SAP. (**A**) a phase contrast image of PBMSCs/SAP scaffold which were induced in osteogenic medium for 21 days. The cells in the scaffold changed its shape into oval-like (arrows). (**B**) ALP staining showing majority of cells in the scaffold were ALP positive. (**b**) an amplified image of Fig. 4B showing lots of ALP positive particles aggregated in some cells (arrows), while other cells have less ALP positive particles scattered in the cytoplasma (arrow heads), which hinted these cells stayed in different stage of differentiation. (**C**) Immunocytochemistry and confocal showing the cells in the scaffold kept in GFP positive (C1) and almost all cells were osteocalcin positive (C2), C3 is the merged image of C1 and C2. (D1-3) After be immunostained with the antibody of chondroitin sulphate proteoglycans (CSPG), the majority of cells were surrounded with immunoreactivities which may aggregated in the scaffold. (c1-3, and d1-3) are local amplified images of C1-3 and D1-3 respectively. Arrows are showing the individual cells, and the arrow heads indicate the interstitial space.

Once mixed with PBMSCs suspension, the solution of SAP was self-assembled into a gel-like 3D scaffold very quickly (about 2 seconds). The cells could keep in surviving and growing in the scaffold when cultured in the osteogenic inductive medium (Fig. 4A). Twenty-one days after induction the scaffold was subjected to be prepared cryosections for further ALP staining and immunocytochemistry. In the ALP stained samples, a lot of ALP positive particles aggregated or scattered in the cytoplasma. The density of these particles were various in different cells (Fig. 4B,b). Immunocytochemistry also showed the cells in the scaffold were immuno-positive for osteocalcin, which is one of the specific markers for osteoblasts and osteocytes(Fig. 4C). Interestingly, when the slides were immunostained with the antibody of CSPG the immuno-reactivity was quite low in the cells but very strong in the scaffold around the cells (Fig. 4D). As CSPG is one of the components of osteoid^35^, this result hinted the induced cells could excrete osteoid-like extracellular matrix and accumulate in the SAP scaffold, which is in favor of new born formation.

### PBMSCs/SAP/PLGA scaffold facilitated the healing of calvarical defect and the new bone formation in the graft

**Figure 5:**
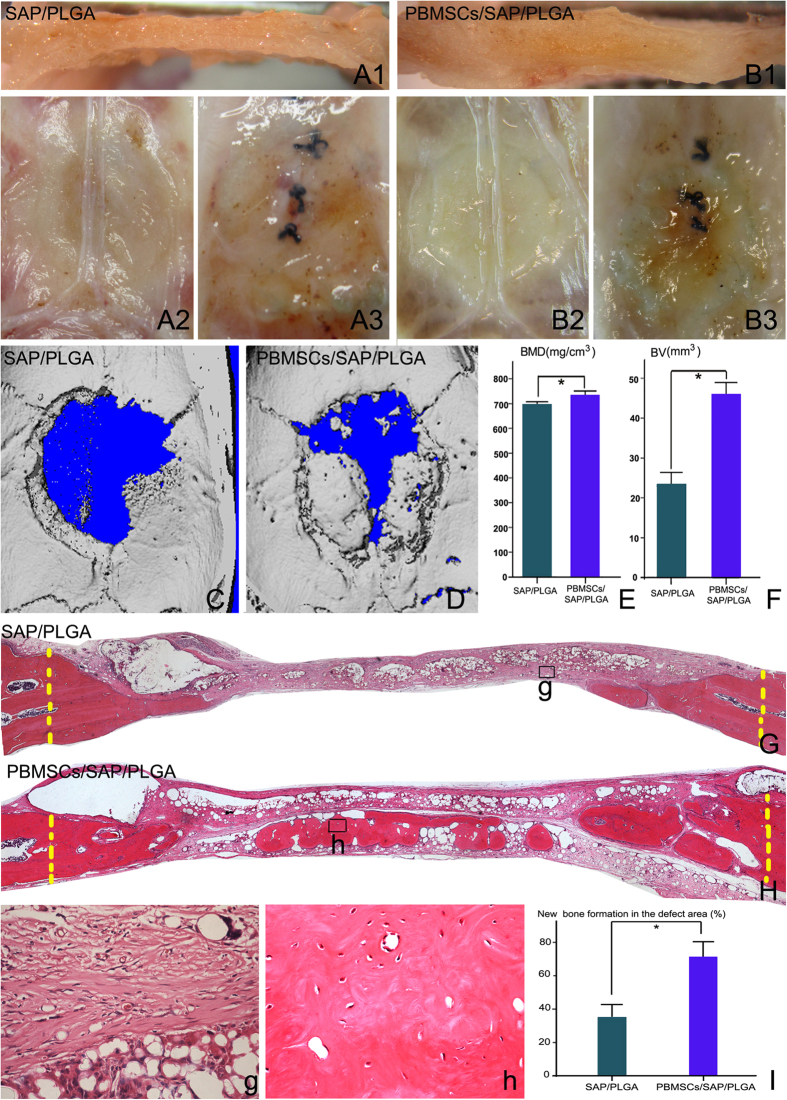
The healing of bony defect and new bone formation. (**A,B**) gross images of the samples harvested at 12 weeks after the cranial defect was transplanted with SAP/PLGA or PBMSCs/SAP/PLGA scaffold. The images of cross-section in the midline of defect area (A1,B1), views of intracranial side (A2,B2) and extracranial side (A3,B3) indicated both of SAP/PLGA and PBMSCs/SAP/PLGA grafts were integrated smoothly with the host. Micro-CT images showing the minerazation in PBMSCs/SAP/PLGA (**C**) and SAP/PLGA (**D**) grafted subjects at 12 weeks post surgery. (**E,F**) Statistical quantifications of the bone mineral density (BMD) and bone volume(BV) in the defect area assessed by micro-CT. (**G,g,H,h**) the whole and local images of H&E staining of cranial defect at 12 weeks post surgery (**G,g**), SAP/PLGA grafted; (**H,h**) PBMSCs/SAP/PLGA grafted), (**I**) Statistical quantifications of the percentage of new formed bone in the defect area assessed by H&E staining.

As designed, the PLGA membranes provided physical support for the inner soft and gel-like scaffold of SAP seeded with or without PBMSCs. Moreover, the membranes are apt to be trimmed to cater to the size of the calvarial defect. Twelve weeks after transplantation the graft was integrated smoothly with the host, regardless if the graft was PBMSCs/SAP/PLGA(Fig. 5A) or SAP/PLGA(Fig. 5B). Micro-CT and 3D reconstruction revealed minerazation was found in the grafted scaffold 12 weeks post surgery. The images showed that the bone formation occurred only around the edge of the calvarical defect in the SAP/PLGA group (Fig. 5C), while the new formed bone filled the majority of the defect area in the PBMSCs/SAP/PLGA group (Fig. 5D). Notably, there were some bony islands scattered in the defect area, which indicated these bony islands resulted from osteogenesis of grafted PBMSCs but not from the osteoblasts of host tissue. In order to quantify the new bone formation in the defects, BV and BMD in the defect area were measured with the built-in software of the Micro-CT system. As showed in Fig. 5E,F, both BV and BMD were significantly higher in PBMSCs/SAP/PLGA group than that of SAP/PLGA group. Micro-CT findings were further confirmed by histological evidence. H&E staining demonstrated the new bone formation mainly localized by the bony borders in the SAP/PLGA group (Fig. 5G,g), while PBMSCs/SAP/PLGA group displayed both peripheral and central distribution (Fig. 5H,h). The difference of the percentage of new bone area quantified from the H&E stained samples was significant between the two groups (Fig. 5I).

### Immunohistological assessments of the survival and osteogenesis of PBMSCs in the SAP nanofiber scaffold

**Figure 6:**
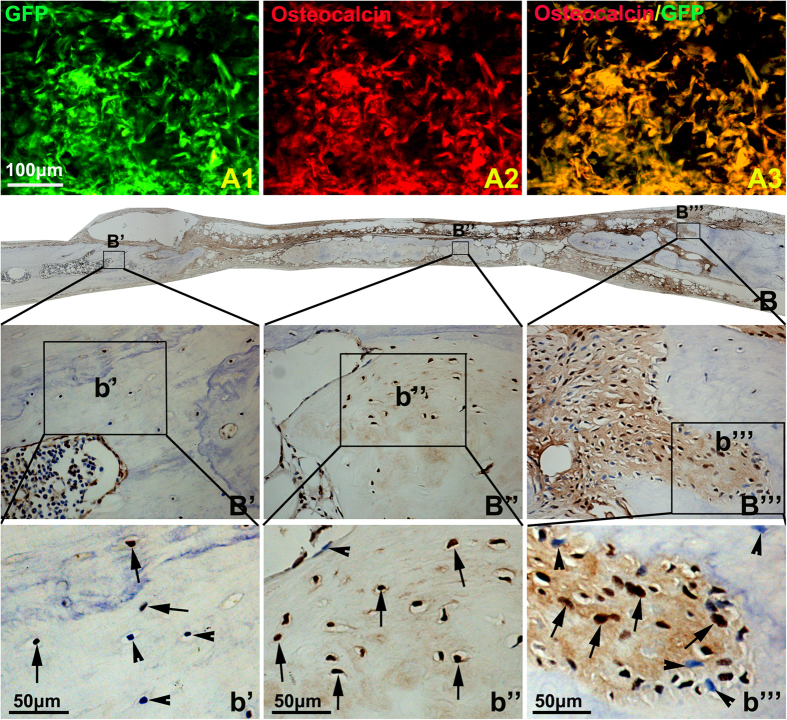
The survival and osteogenesis of grafted PBMSCs in the bony defect area. (**A**) Lots of GFP positive graft cells were detected in the bony defect area 2 weeks post-implantation (A1), and these cells were positive for osteocalcin (A2), A3 is the merged image of A1 and A2. (**B**) BrdU immunohistochemistry showing lots of BrdU labeled graft cells were embedded by new formed bone matrix. In order to show the details in different areas, local images were step by step amplified and showed in (**B’–B”’**) and (**b’–b”’**). In the central area, almost all of the osteocytes in the new formed bone were BrdU positive (arrows),while in the edge of defect area, both of BrdU positive (arrows) and negative (arrow heads) osteocytes were located in the new formed bone.

Even though both micro-CT and H&E staining showed the PBMSCs/SAP/PLGA scaffold promoting the new bone formation in the cranial defect, it still cannot declare that PBMSCs survived in the grafted scaffold and contributed to the new bone formation. At 2 weeks post-implantation, immunohistochemistry and confocal microscopy revealed plenty of GFP positive cells survived within the grafts of PBMSCs/SAP/PLGA scaffolds, while the osteocalcin immunoreactive signal was overlapped with majority of the GFP positive cells (Fig. 6A). The above data strongly support the transplanted PBMSCs could survive and differentiate into osteoblasts in the bony defect area. Moreover, BrdU assay performed at 12 weeks post-implantation showed that a majority of osteoctyes embedded in the new formed bone were positive for BrdU (Fig. 6B). This meant the cells in this area were derived from the PBMSCs and these cells directly contribute to form new bone matrix in the defect area.

## Discussion

The initial question addressed by present study is the capability of PBMSCs in osteogenesis and new bone formation. Secondly, we aimed to fabricate a novel tissue engineering scaffold with PBMSCs/SAP/PLGA to reconstruct the large scale cranial defects.

Utilizing a FCM assay, the immunophenotyping of the harvested cells from peripheral blood showed more than 97% cells were positive for CD29d, CD44, CD90,CD105, and CD146 which are the widely accepted markers of mesenchymal stem cells^7^. On the contrary, less than 0.1% cells were positive for hematopoietic lineage marker CD45^7^. Moreover, we successfully induced these cells into multilineage cells, including osteoblasts, chondroblasts, adipocytes, neurons and Schwann cells. Both of the immunophenotyping and multidifferentiation ability identified by the present study, draws us to believe these cells have characters of mesenchymal stem cells. Therefore, we named it PBMSCs as previously reported^11^,^13^. Since the attractive potential, PBMSCs are already used to repair tissue injuries including bone defects. Wang *et al*.^13^ seeded PBMSCs into a porous calcium phosphate scaffold to treat a 20 mm length ulna bone defect in rabbit. Their main results were the following: X-ray assay showed mineralization in the PBMSCs seeded scaffold was better than the scaffold only. H&E staining indicated small amount of new bone was formed in PBMSCs seeded scaffolds. Very recently, Lorenz’s group reported a study of using PBMSCs seeded hydroxyapatite- poly(lactic-coglycolic acid) (HA-PLGA) to repair a 4 mm calvarial bone defect. 2, 4, 6 and 8 weeks post-surgery, the healing of bone defect was evaluated by micro-CT^14^. Again, their data also supported PBMSCs can enhance new bone formation. However, both of the above publications did not mention the survival and osteogenesis of PBMSCs. Can the cells survive in the grafts? Is the new bone formation contributed from the grafted PBMSCs? There are still critical questions. Data from the present study, to our knowledge, is the first time to provide solid evidence to answer these questions. The GFP and BrdU signals detected in the implants 2 or 12 weeks post-surgery are accepted standard for labeling grafted cell *in vivo*. Considering the information of osteocalcin immunoreactivities overlapped with GFP signals and BrdU positive cells embedded with new formed bone matrix, a consolidated conclusion is drawn that the transplanted PBMSCs could survive in the scaffold *in vivo* and contribute to new bone formation. This means that PBMSCs could serve as a new cell source for bony tissue engineering.

To mimic the special structural and functional features of cranial bone, we designed a sandwich like scaffold to repair the calvarial defect, which was composed of two PLGA membranes in double sides and PBMSCs seeded SAP inside. PLGA is a FDA approved degradable biomaterial and is widely used for clinic tissue repair^36^,^37^. The PLGA membranes could seal the bony defect hole to avoid the brain tissue being exposed to outer hazards and provide mechanical support for the soft gel-like SAP inside, which is an optimal scaffold for the PBMSCs survival and osteogenesis to accelerate the new bone formation. The PLGA membranes were also fabricated with porosity as described in methods. We suppose the porosity is conducive for nutrients and metabolites to pass through the membrane and may good for the survival of the grafted cells within the scaffold.

Nanofiber scaffolds are novel attracting biomaterials which have been widely adopted for kinds of tissue engineering recently^38^−^40^. We, herein, used RADA16-I, a type of self-assembling peptides (SAP), to prepare the composited scaffold with PLGA and PBMSCs. SAP belongs to a special class of ionic-complementary peptides that consist of alternating hydrophilic and hydrophobic amino acid residues. Once introduced into solutions of electrolytes, the peptides can self assemble into well-ordered nanofiber scaffolds with a lot of advantages in topography and porosity, which provide excellent physiological compatibility, plasticity, and a true 3-D microenvironment similar to natural extracellular matrix^41^. Our previous studies indicated SAP has properties to reconstruct the injured nervous system, and serve as an effective delivery system for graft cells or therapeutic drugs^23^,^33^,^38^,^42^. The present data also demonstrats that the SAP scaffold effectively facilitates PBMSCs survival both *in vitro* and *in vivo*. Also, after the cells were differentiated into osteoblasts or osteocytes, their secretion (e.g. CSPG) might be aggregated in the SAP which may result in new osteoid and further bone matrix formation^35^. This may helpful to understand the findings of CSPG immunoreactivities surrounding the PBMSCs (Fig. 4D) and the new formed bone matrix embedding the BrdU positive grafted cells *in vivo* (Fig. 6B).

## Conclusions

The current study demonstrated that a type of mesenchymal stem cells are existing in the peripheral blood. These cells can be induced into multilineage cells, including osteoblasts. After be seeded into a three dimensional nanofiber scaffold of SAP, the cells survive and undergo osteogenesis both *in vitro* and *in vivo*. We also fabricated a composited tissue engineering scaffold made of PBMSCs, SAP and PLGA, and used it to repair a 8 mm cranial defect which is a typical calvarial critical size bone defect model in rat^34^. To our knowledge, this is the first report of solid evidence to demonstrate that the transplanted PBMSCs can survive in the bone defect area and can directly contribute to new bone formation. Moreover, the present data also indicated that the tissue engineering with PBMSCs/SAP/PLGA scaffold can serve as a novel prospective strategy for healing large size cranial defects.

## Additional Information

**How to cite this article**: Wu, G. *et al*. Osteogenesis of peripheral blood mesenchymal stem cells in self assembling peptide nanofiber for healing critical size calvarial bony defect. *Sci. Rep*. **5**, 16681; doi: 10.1038/srep16681 (2015).
